# The Influence of Poverty and Rurality on Colorectal Cancer Survival by Race/Ethnicity: An Analysis of SEER Data with a Census Tract-Level Measure of Persistent Poverty

**DOI:** 10.3390/curroncol32050248

**Published:** 2025-04-23

**Authors:** Steven S. Coughlin, Meng-Han Tsai, Jorge Cortes, Malcolm Bevel, Marlo Vernon

**Affiliations:** 1 Department of Biostatistics, Data Science and Epidemiology, Augusta University, Augusta, GA 30912, USA; 2 Georgia Prevention Institute, Augusta University, Augusta, GA 30912, USA; 3 Cancer Prevention, Control, & Population Health Program, Georgia Cancer Center, Augusta University, Augusta, GA 30912, USA; 4 Georgia Cancer Center, Augusta University, Augusta, GA 30912, USA

**Keywords:** income, poverty, race, rurality, social determinants of health

## Abstract

**Purpose:** Because of shared mechanisms such as decreased access to health care, rurality and poverty may act synergistically to decrease colorectal cancer (CRC) survival. **Methods:** We conducted a retrospective cohort analysis of SEER data (22 registries) with census tract-level measures of poverty/rurality for the period 2006–2015. Multivariable Cox proportional hazard regressions were applied to examine the independent and intersectional associations of persistent poverty and rurality on 5-year cause-specific CRC survival across five racial/ethnic groups. **Results:** Among 532,868 CRC patients, non-Hispanic Blacks (NHB) demonstrated lower 5-year survival probability (64.2% vs. 68.3% in non-Hispanic Whites [NHW], 66.5% in American Indian/Alaska Natives [AI/AN], 72.1% in Asian/Pacific Islanders, and 68.7% in Hispanic groups) (*p*-value < 0.001). In adjusted analysis, CRC patients living in rural areas with poverty were at a 1.2–1.6-fold increased risk of CRC death than those who did not live in these areas in five racial/ethnic groups. In particular, AI/AN patients living in rural areas with poverty were 66% more likely to die from CRC (95% CI, 1.32, 2.08). **Conclusions:** CRC patients who live in rural or poverty areas in SEER areas in the U.S. have a poorer survival compared with those who do not live in such areas regardless of race/ethnicity. Significantly greater risk of CRC death was observed in AI/ANs. **Impact:** Patient navigators, community education or screening, and other health care system interventions may be helpful to address these disparities by socioeconomic status, race, and geographic residence. Multi-level interventions aimed at institutional racism and medical mistrust may also be helpful.

## 1. Introduction

Colorectal cancer (CRC) is the third leading cause of cancer-related death among individuals in the U.S. [1,2]. Socioeconomic factors such as household income are well established factors associated with CRC mortality patterns [3]. Several studies have shown that low income is associated with an increased risk of poorer survival [4,5,6,7,8]. Disparities in CRC survival by socioeconomic status may be due to a lack of health insurance, lack of transportation, and other structural factors [9,10].

Rural residence has also been associated with increased risk of CRC mortality [11,12,13]. Several factors may explain the disparities in CRC survival by rural residence, including a later stage at diagnosis and lesser access to specialized centers and clinical trials [11]. Compared to urban areas, rural communities face higher poverty rates, lower educational attainment, a lack of transportation, and a lack of access to health care services [14,15]. Shared mechanisms such as decreased access to health care, rurality, and poverty may also act synergistically to decrease CRC survival.

Significant disparities in colorectal cancer mortality exist in the U.S. according to race and Hispanic ethnicity. For example, colorectal cancer mortality rates are 44% higher in Black men and 31% higher in Black women than in Whites [2]. Racial and ethnic disparities in colorectal cancer mortality may be due to several factors including decreased access to health care services, institutional racism, and medical mistrust [16,17,18]. Prior research has shown that people in some racial and ethnic groups are less likely to receive a recommendation for CRC screening from their doctor [19].

To further explore these associations, we analyzed SEER data with a census tract-level measure of persistent poverty for the period 2006–2015. We extended previously reported studies by using more recent data and by examining the independent and intersectional associations of persistent poverty and rurality with 5-year cause-specific CRC survival across five racial/ethnic groups (non-Hispanic White [NHW], non-Hispanic Black [NHB], American Indian/Alaska Native [AI/AN], Asian/Pacific Islander [PI], and Hispanic). We also compared the 5-year survival rates by persistent poverty and rurality within these five racial/ethnic groups. Such research is vital for informing interventions tailored to specific racial/ethnic groups, who live in low-resource communities.

## 2. Materials and Methods

### 2.1. Study Design and Data Sources

We conducted a retrospective cohort analysis using data from the 2006–2015 Incidence Data with Census Tract Attributes from Surveillance, Epidemiology, and End Results (SEER) Program (22 registries, November 2022 submission). SEER data include patient demographics, primary tumor site, tumor morphology and stage at diagnosis, first course of treatment, and vital status, as well as information on persistent poverty and rurality. The eligible study population included patients diagnosed with CRC as defined by the SEER Site Recode ICD-O-3/WHO 2008 definition of colon cancer (C180–C189), rectosigmoid junction cancer (C199), and rectal cancer (C209). The extracted data were publicly available, de-identified, and thus exempt from institutional review board (IRB) review. A STROBE statement for cohort studies is included in the Appendix A.

### 2.2. Study Participants

A total of 835,907 CRC patients were included in the SEER program. To obtain a more homogeneous eligible study sample, we excluded CRC patients aged under 20 years (n = 1600), duplicate records of the same patients (n = 11,338), missing information on rurality (n = 22) and persistent poverty (n = 248), CRC diagnosed after 2015 due to limited follow-up time (less than five years) (n = 275,291), missing survival time (n = 5913), missing vital status (n = 18), missing race (n = 2095), and missing information on surgery (n = 6514). A five-year interval, which is a common indicator for cancer survival research, was used as one of the sample selection criteria because it provides a consistent follow-up time for all patients. These sample selections were based on prior publications that focused on CRC mortality using the SEER program. As a result, 532,868 eligible CRC patients were included in the full analyses.

### 2.3. Measures: Outcome, Exposure, and Covariates

Cause-specific CRC survival was the outcome of interest, and race/ethnicity (NHW, NHB, AI/AN, Asian/PI, and Hispanic), persistent poverty (yes or no), and rurality (yes or no) were our exposures of interest. Both the definition of poverty and rurality were incorporated at the time of cancer diagnosis. The definition of persistent poverty was based on 1990 and 2000 decennial censuses and 2007–2011 and 2015–2019 American Community Survey 5-year estimates, and it was defined as when 20% or more of the population has lived below the poverty level for a period spanning about 30 years. Poverty information may be mismatched due to time differences in the information. Further, rurality was defined by using the 2010 U.S. Department of Agriculture’s (USDA’s) Rural Urban Commuting Area (RUCA) codes. RUCA codes 1.0, 1.1, 2.0, 2.1, 3.0, 4.1, 5.1, 7.1, 8.1, and 10.1 were considered as urban areas (termed as no) and all other codes were classified as rural areas (termed as yes). Further, we defined poverty and rurality as a four-category variable (poverty alone, rurality alone, poverty and rural, and non-poverty and rural), which are not mutually exclusive categories. Other covariates of interest were demographic characteristics, tumor characteristics, and initial treatment modalities. Those covariates were adjusted in multivariable models and evaluated for their impact on cause-specific CRC survival. Demographic characteristics included age at diagnosis (20–39, 40–59, 60–79, or 80+ years), gender (male or female), marital status (single, married, others, or unknown), and year of diagnosis (2006–2010 or 2011–2015). We also included tumor grade (grade 1, 2, 3, 4, or unknown), SEER summary stage at diagnosis (localized, regionalized, distant, or unknown), and primary site (right or left). Tumor grade and stage at diagnosis were included as ordinal categorical variables with grade 4 and distant disease considered as advanced diagnoses. The patients’ initial treatment modality, surgery (yes or no), chemotherapy (yes or no/unknown), and radiation (yes or no/unknown) were also included.

### 2.4. Statistical Analysis

Descriptive statistics were used to describe the distribution of CRC patients according to persistent poverty, rurality, demographic characteristics, tumor characteristics, and initial treatment modality. We compared bivariate differences in poverty, rurality, demographic characteristics, tumor characteristics, and initial treatment modalities across five racial/ethnic groups using the chi-square test for categorical variables. Survival analysis was performed using the Kaplan–Meier method. The log-rank test was used to compare the survival rates within persistent poverty and rurality across different racial and ethnic groups. Further, we performed Cox proportional hazard regression to examine the impact of race/ethnicity, persistent poverty, and rurality on 5-year cause-specific CRC survival in the US. Four sequential models were developed to examine this association. The crude model included race/ethnicity, persistent poverty, and rurality. Model 1 was further adjusted for demographic characteristics (age at diagnosis, gender, race, marital status, and year of diagnosis); model 2 was further adjusted for tumor characteristics (grade, SEER summary stage, primary site); and model 3 was further adjusted for initial treatment modality (surgery, radiation, chemotherapy). Additional analysis was performed to further examine the mentioned association when excluding tumor grade and SEER summary stage at diagnosis were unknown to reduce the concerns regarding unknown information being included. Finally, we examined the interaction between persistent poverty and rurality on cause-specific CRC survival across five racial/ethnic groups, adjusting for demographic characteristics, tumor characteristics, and initial treatment modality. We measured CRC patients’ survival time in months from the date of diagnosis up to 60 months of follow-up, censored at the end of the study observation period (31 December 2020) or death due to all other causes. The goal of this study was to report 5-year cause-specific mortality and use approaches appropriate for the 5-year binary outcome. The use of traditional methods (e.g., log-rank or Cox models) provides the relative risk of the event of interest. To elucidate potential bias, we also performed competing risk models to evaluate whether race/ethnicity, rurality, and persistent poverty were associated with CRC death.

The level of statistical significance was set at an alpha level of 0.05, and *p*-values were based on two-sided probability tests. We used SAS Version 9.4, SAS Institute Inc., Cary, NC, USA, and SPSS version 28.0 to perform analyses.

### 2.5. Data Availability

The datasets generated during the current study are available in the Surveillance, Epidemiology, and End Results Program (data can be requested through https://seer.cancer.gov/).

## 3. Results

### 3.1. Patient Demographics, Tumor Characteristics, and Initial Treatment Modality

As shown in Table 1, the majority of CRC patients lived in non-persistent poverty (89.5%) and non-rural (86.2%) areas. Many were aged 60–79 years (47.6%), male (52.1%), married (42.7%), diagnosed with CRC during 2006–2010 (50.6%), had a grade 2 diagnosis (57.3%), localized disease (39.2%), left-sided CRC (59.2%), receipt of surgery (81.7%), no radiation therapy (89%), or no chemotherapy (65.1%). When examining racial/ethnic differences, a sizeable percentage of patients living in persistent-poverty areas were NHB (27.9%), followed by Hispanic (23%) (*p*-value < 0.001). In contrast, 29.1% of the patients living in rural areas were AI/AN, followed by NHW (16.8%) and NHB (8.5%) (*p*-value < 0.001).

### 3.2. Five-Year Survival Probability

**Figure 1:**
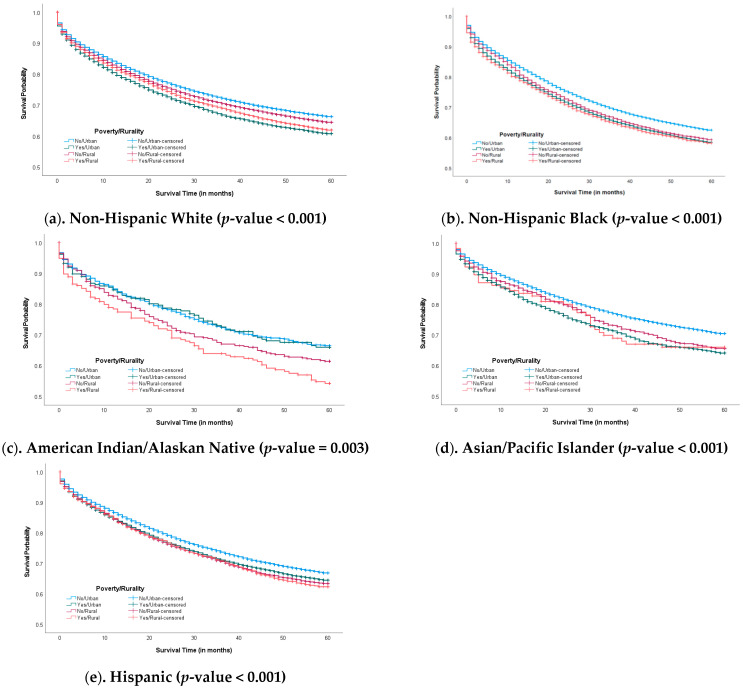
Kaplan–Meier colorectal cancer survival curves. Survival by poverty and rurality. In Figure 1, colorectal cancer survival was different when comparing poverty and rurality across different race/ethnicity groups.

The mean duration of follow-up time since CRC diagnosis was 40.9 months (SD, 23.4 months). The overall five-year survival rate was 68.1% among CRC patients in the U.S. Based on race and ethnicity, the corresponding probabilities were 68.3% for NHW, 64.2% for NHB, 66.5% for AI/AN, 72.1% for Asian/PI, and 68.7% for Hispanic groups (*p*-value < 0.001). Further, we used the KM approach to evaluate the PH assumption in our three exposures of interest (race/ethnicity, rurality, poverty) separately. The survival curves followed similar trends without crossing, which met the PH assumption [20]. When exploring the survival probability according to persistent poverty and rurality, the five-year survival probability for those living in urban areas with persistent poverty was 64.6% (vs. 65.1% in rural/persistent poverty, 67.1% in rural but not persistent poverty, 68.8% in urban but not persistent poverty, respectively; *p*-value < 0.001) among NHW (Figure 1a) and 67.6% (vs. 67.8% in rural/persistent poverty, 68.2% in rural but not persistent poverty, 72.6% in urban but not persistent poverty, respectively; *p*-value < 0.001) among Asian/PI patients (Figure 1d). In Figure 1b,c,e the survival curves overlapped somewhat. Among non-NHW patients, those living in rural and persistent-poverty areas had a lower 5-year survival probability than those living in areas with persistent poverty alone, rurality alone, or neither.

### 3.3. Association Between Race/Ethnicity, Persistent Poverty, Rurality, and Cause-Specific CRC Survival

As shown in Table 2, results from the four models were similar when adjusting for different covariates. In the full model, we found that NHB patients had a 1.1-fold increased risk of CRC death (hazard ratio [HR], 1.12; 95% CI, 1.10–1.13) in comparison to NHW patients. However, Asian/PI and Hispanic patients were 9% (HR, 0.91; 95% CI, 0.89–0.93) and 3% (HR, 0.97; 95% CI, 0.96–0.99) less likely to die from CRC, respectively, compared to NHW patients. More importantly, CRC patients living in areas designated as persistent poverty (HR, 1.10; 95% CI, 1.08–1.12) and rural (HR, 1.11; 95% CI, 1.10–1.13) had a 1.1-fold increased risk of CRC death compared with those who did not live in these areas. Similar results were also observed from competing risk models. In the full adjusted analysis, NHB patients were 1.1-fold more likely to die from CRC (HR, 1.1; 95% CI, 1.09–1.12). Asian/PI and Hispanic patients were 15% (HR, 0.85; 95% CI, 0.83–0.87) less likely to die from CRC; however, no significant difference was observed for Hispanic patients. CRC patients living in areas designated as persistent poverty (HR, 1.06; 95% CI, 1.05–1.08) and rural (HR, 1.09; 95% CI, 1.07–1.10) also had 1.1-fold increased risk of CRC death. Finally, we examined the association when adjusting for all covariates by excluding unknown tumor grade and SEER summary stage. Results from the full model and reduced model were similar (Supplementary Table S1).

As shown in Table 3, CRC patients living in persistent poverty and rural areas had a 1.2–1.6-fold increased risk of CRC death compared to those living in non-persistent poverty and non-rural areas (NHW: HR, 1.26, 95% CI, 1.21–1.31; NHB: HR, 1.2, 95% CI, 1.12–1.28; AI/AN: HR, 1.66, 95% CI, 1.32–2.08; Hispanic: HR, 1.18, 95% CI, 1.09–1.29, all *p*-values < 0.001). A 66% higher risk of cause-specific death for CRC was observed in AI/ANs. Higher estimate was also observed for AI/ANs when conducting a competing risk model (HR, 1.45; 95% CI, 1.08–1.94). Further, CRC patients living in non-rural, persistent-poverty areas were about 11% more likely to die from CRC for NHWs, NHBs, Asian/PIs, and Hispanics (NHW: HR, 1.13, 95% CI, 1.10–1.17; NHB: HR, 1.10, 95% CI, 1.07–1.14; AI/AN: HR, 1.11, 95% CI, 1.03–1.19; Hispanic: HR, 1.05, 95% CI, 1.01–1.09, all *p*-values < 0.001). Similarly, we found that CRC patients living in rural, non-persistent-poverty areas were about 1.1–1.2-times more likely to die from CRC for NHWs, NHBs, Asian/PIs, and Hispanics (NHW: HR, 1.10, 95% CI, 1.08–1.12; NHB: HR, 1.11, 95% CI, 1.05–1.18; AI/AN: HR, 1.17, 95% CI, 1.05–1.31; Hispanic: HR, 1.15, 95% CI, 1.09–1.22, all *p*-values < 0.05).

## 4. Discussion

To our knowledge, this is the first study to examine the intersectional associations between persistent poverty and rurality and cause-specific CRC survival using the SEER incidence data with census tract attributes. We found that CRC patients living in rural areas or areas with persistent poverty had a slightly increased risk of CRC death compared to those not living in a rural or persistent-poverty area. When examining racial/ethnic differences, regardless of racial/ethnic groups, CRC patients living in rural areas with persistent poverty were at a 1.2–1.6-fold increased risk of CRC death, with a higher estimate for AI/ANs. Findings from our study indicate that the intersectional associations between persistent poverty and rurality may be an important determinant of cause-specific CRC survival when considering diverse racial/ethnic groups.

Overall, the results of this study indicate that CRC patients living in persistent poverty and rural areas have an increased risk of CRC death compared to those not living in these areas regardless of race/ethnicity. Several factors may account for this finding, including a lack of access to quality health care, a decreased supply of health care providers, a lack of transportation, and institutional racism [15,21]. Access to timely quality care is an important mediator of CRC mortality [11]. Patient factors such as medical distrust or health illiteracy may also play a role. Compared to urban areas, rural communities have higher poverty rates, lower educational attainment, a lack of transportation, and a lack of access to health care services [14,15]. The disparities in CRC survival may be partly due to a lack of colonoscopy services in rural and persistent-poverty areas [11]. Social determinants of health (e.g., poverty, rurality, food deserts, food swamps) are complex and interconnected, requiring a more comprehensive approach to analyze and intervene. Possible solutions must consider the entire spectrum of social factors that influence outcomes across the cancer continuum (primary prevention, early detection, treatment, and survivorship). This may be particularly true for AI/ANs as we found that AI/AN patients living in persistent poverty and rural areas had a higher risk of CRC mortality. Lower awareness of CRC among AI men may explain the CRC mortality disparities in these groups [20]. Because of limited research on this topic, more studies are needed to elucidate the specific barriers that impact CRC outcomes among AI/ANs.

When examining 5-year survival probability across different racial/ethnic groups, Black patients with CRC had the worst 5-year survival of any of the five racial/ethnic groups we examined, which is consistent with the prior literature [1]. In an analysis of data from the National Cancer Database from 2004 to 2018, Tobin et al. [12] found that both rurality and Black race negatively affect the survival of patients with stage II-III CRC and that they may act synergistically to worsen outcomes. Moss et al. [21] found that Black residents of rural, persistent-poverty counties had the highest mortality rates for six out of eight cancer outcomes. We found that NHB patients who live in rural areas with persistent poverty were at a 1.2-fold increased risk of CRC death compared with those who did not live in these areas. Several factors may account for this disparity, including a lack of access to timely and quality care, differences in disease management and treatment, cultural factors, discrimination, medical distrust, and provider bias [7,22].

The associations between living in a persistent-poverty area and decreased CRC survival may differ according to race and ethnicity, as found in the literature [5]. Barriers to access to care, including financial barriers, environmental barriers such as lack of transportation and nearby facilities, and social environment barriers such as social and cultural factors, may explain these disparities [7].

Social norms, such as community standards, group expectations, common practices, and shared beliefs, can also influence a community’s intention to access cancer screening and treatment. In previous research, sociodemographic differences observed in CRC screening were mediated through social cognitive factors, supporting the findings of this project for a multi-dimensional approach that includes addressing social norms [23].

The strengths of the current study include the large sample size, geographic coverage, and the availability of a census tract-level measure of persistent poverty as well as rurality. In particular, we considered the intersectional associations between persistent poverty and rurality and cause-specific CRC survival, which may reflect the actual impact of health resources on cause-specific survival for CRC. Our findings are critical to inform health system policies for health-related investments in rural and persistent-poverty areas. Health resources may include specialized health care professionals and screening facilities to promote early detection and treatment of CRC [24].

With respect to the generalizability of the current study findings, our results may be applicable to other population groups with similar structural characteristics (e.g., persistent poverty, rural residence, limited access to health care, systemic marginalization). The current study should be replicated in other geographic and sociocultural contexts—such as Indigenous communities in Canada or rural areas of Central and Eastern Europe. This would allow for the verification of the broader validity of the findings and support the development of relevant public health strategies aimed at reducing inequalities in cancer survival. With respect to other limitations, first, individual-level data for lifestyle factors were not available as cancer registries usually do not collect this information. For example, physical inactivity, obesity, current smoking behaviors, pro-inflammatory dietary habits, and heavy alcohol use may greatly increase the risk of CRC mortality [3]. The absence of these covariates in SEER data may have resulted in residual confounding which could bias risk estimates. The presence of chronic disease conditions was also not available for evaluation because of unavailable information from the SEER program. Second, ecological fallacy is a possibility because of the use of census tract-level measures of persistent poverty and rurality. Additional information about structural barriers related to poverty or rurality at the individual and community-level (e.g., lack of transportation, lack of treatment or screening facilities) may be needed to further elucidate CRC mortality. However, that information was not available in the SEER program. Evidence has shown that addressing structural barriers at different levels may enhance CRC survival through timely cancer care [25]. More research integrating the multifactorial factors is needed to further elucidate the relationship between our findings and CRC survival in rural areas with persistent poverty. Fourth, although initial treatment was included as covariates, the analysis did not explore differences in treatment quality, timing, or completion. Fifth, poverty and rurality information may mismatch due to time differences in the information, which may have overestimated the effect of poverty and/or rurality on cause-specific survival for CRC. The definition of poverty and rurality may also vary across different timeframes. Thus, more research using different timeframes of poverty and rurality definition may be helpful. Finally, although treatment modality is not our exposure of interest, future research using this factor should be cautious due to the completeness of the variables. Future research examining the intersecting relationship of poverty and rurality should consider the stage at diagnosis in greater detail.

## 5. Conclusions

In conclusion, CRC patients who live in a rural or persistent-poverty area in SEER areas in the U.S. have poorer cause-specific survival for CRC compared with those who do not live in such areas regardless of racial/ethnic groups, with significant higher risk for AI/AN patients. Patient navigators, community education on screening, access to various screening opportunities, and health care system interventions may be helpful in addressing these disparities by socioeconomic status, race, Hispanic ethnicity, and geographic residence [3]. Policies that increase the supply of health care providers in rural and persistent-poverty areas may also help to alleviate these disparities. In particular, multi-level interventions are needed to alleviate these disparities in cause-specific CRC survival [21].

## Figures and Tables

**Table 1:** Characteristics of study population. Distribution of persistent poverty, rurality, demographic characteristics, tumor features, and treatment modality by race/ethnicity.

	Total (n = 532,868)	NHW (n = 363,505)	NHB (n = 63,537)	AI/AN (n = 2236)	Asian/PI (n = 35,453)	Hispanic (n = 68,137)	*p*-Value
	**n (%)**						
**Persistent poverty** ^a^							<0.001
No	476,874 (89.5%)	343,676 (94.6%)	45,843 (72.2%)	1782 (79.7%)	33,113 (93.4%)	52,460 (77.0%)	
Yes	55,994 (10.5%)	19,829 (5.5%)	17,694 (27.9%)	454 (20.3%)	2340 (6.6%)	15,677 (23.0%)	
**Rurality** ^a^							<0.001
No	459,119 (86.2%)	302,279 (83.2%)	58,171 (91.6%)	1585 (70.9%)	34,273 (96.7%)	62,811 (92.2%)	
Yes	73,749 (13.8%)	61,226 (16.8%)	5366 (8.5%)	651 (29.1%)	1180 (3.3%)	5326 (7.8%)	
**Poverty/Rurality**							<0.001
Poverty alone	43,550 (8.2%)	11,749 (3.2%)	15,376 (24.2%)	238 (10.6%)	2222 (6.3%)	13,965 (20.5%)	
Rural alone	61,305 (11.5%)	53,146 (14.6%)	3048 (4.8%)	435 (19.5%)	1062 (3.0%)	3614 (5.3%)	
Poverty & Rural	12,444 (2.3%)	8080 (2.2%)	2318 (3.7%)	216 (9.7%)	118 (0.3%)	1712 (2.5%)	
Non-Poverty & Rural	415,569 (78.0%)	290,530 (79.9%)	42,795 (67.4%)	1347 (60.2%)	32,051 (90.4%)	48,846 (71.7%)	
**Demographic characteristics**							
**Age**							<0.001
20–39 years	15,506 (2.9%)	8412 (2.3%)	1990 (3.1%)	107 (4.5%)	1317 (3.7%)	3680 (5.4%)	
40–59 years	147,500 (27.7%)	88,534 (24.4%)	22,665 (35.7%)	773 (34.6%)	11,543 (32.6%)	23,985 (35.2%)	
60–79 years	253,664 (47.6%)	174,944 (48.1%)	30,225 (47.6%)	1097 (49.1%)	16,537 (46.6%)	30,861 (45.3%)	
80+ years	116,198 (21.8%)	91,615 (25.2%)	8657 (13.6%)	259 (11.6%)	6056 (17.1%)	9611 (14.1%)	
**Gender**							<0.001
Male	277,537 (52.1%)	189,128 (52.0%)	31,499 (49.6%)	1157 (51.7%)	18,520 (52.2%)	37,233 (54.6%)	
Female	255,331 (47.9%)	174,377 (48.0%)	32,038 (50.4%)	1079 (48.3%)	16,933 (47.8%)	30,904 (45.4%)	
**Marital status**							<0.001
Single	69,090 (13.0%)	39,019 (10.7%)	15,307 (24.1%)	417 (18.7%)	4317 (12.2%)	10,030 (14.7%)	
Married	227,687 (42.7%)	163,430 (45.0%)	18,614 (29.3%)	868 (38.8%)	20,695 (58.4%)	24,080 (35.3%)	
Other ^b^	122,132 (22.9%)	90,017 (24.8%)	14,268 (22.5%)	546 (24.4%)	6433 (18.2%)	10,868 (16.0%)	
Unknown	113,959 (21.4%)	71,039 (19.5%)	15,348 (24.2%)	405 (18.1%)	4008 (11.3%)	23,159 (34.0%)	
**Year of diagnosis**							<0.001
2006–2010	269,396 (50.6%)	188,606 (51.9%)	31,641 (49.8%)	972 (43.5%)	16,760 (47.3%)	31,417 (46.1%)	
2011–2015	263,472 (49.4%)	174,899 (48.1%)	31,896 (50.2%)	1264 (56.5%)	18,693 (52.7%)	36,720 (53.9%)	
**Tumor features**							
**Grade** ^c^							<0.001
Grade 1	47,731 (9.0%)	31,022 (8.5%)	6472 (10.2%)	206 (9.2%)	3136 (8.9%)	6895 (10.1%)	
Grade 2	305,250 (57.3%)	208,444 (57.3%)	35,787 (56.3%)	1283 (57.4%)	21,136 (59.6%)	38,600 (56.7%)	
Grade 3	79,249 (14.9%)	57,303 (15.8%)	7613 (12.0%)	284 (12.7%)	4740 (12.4%)	9309 (13.7%)	
Grade 4	10,285 (1.9%)	7841 (2.2%)	882 (1.4%)	40 (1.8%)	506 (1.4%)	1016 (1.5%)	
Unknown	90,353 (17.0%)	58,895 (16.2%)	12,783 (20.1%)	423 (18.9%)	5935 (16.7%)	12,317 (18.1%)	
**SEER Summary Stage**							<0.001
Localized	208,901 (39.2%)	144,787 (39.8%)	24,077 (37.9%)	821 (36.7%)	13,969 (39.4%)	25,247 (37.1%)	
Regionalized	185,436 (34.8%)	127,683 (35.1%)	20,304 (32.0%)	772 (34.5%)	12,705 (35.8%)	23,972 (35.2%)	
Distant	108,900 (20.4%)	71,773 (19.7%)	15,229 (24.0%)	501 (22.4%)	6788 (19.2%)	14,609 (21.4%)	
Unknown	29,631 (5.6%)	19,262 (5.3%)	3927 (6.2%)	142 (6.4%)	1991 (5.6%)	4309 (6.3%)	
**Primary site** ^d^							<0.001
Right	217,640 (40.8%)	155,220 (42.7%)	26,871 (42.3%)	801 (35.8%)	10,514 (29.7%)	24,234 (35.6%)	
Left	315,228 (59.2%)	208,285 (57.3%)	36,666 (57.7%)	1435 (64.2%)	24,939 (70.3%)	43,903 (64.4%)	
**Treatment modality**							
**Surgery**							<0.001
No	97,699 (18.3%)	63,682 (17.5%)	13,892 (21.9%)	473 (21.2%)	5971 (16.8%)	13,681 (20.1%)	
Yes	435,169 (81.7%)	299,823 (82.5%)	49,645 (78.1%)	1763 (78.9%)	29,482 (83.2%)	54,456 (80.0%)	
**Radiation**							<0.001
No	474,252 (89.0%)	323,109 (88.9%)	57,807 (91.0%)	1866 (83.5%)	30,577 (86.3%)	60,893 (89.4%)	
Yes	58,616 (11.0%)	40,396 (11.1%)	5730 (9.0%)	370 (16.6%)	4876 (13.8%)	7244 (10.6%)	
**Chemotherapy**							<0.001
No	346,719 (65.1%)	239,971 (66.0%)	41,370 (65.1%)	1332 (59.6%)	22,145 (62.5%)	41,901 (61.5%)	
Yes	186,149 (34.9%)	123,534 (34.0%)	22,167 (34.9%)	904 (40.4%)	13,308 (37.5%)	26,236 (38.5%)	

Abbreviations: CRC, colorectal cancer; NHW, non-Hispanic White; NHB, non-Hispanic Black; AI/AN, American Indian/Alaska Native; PI, pacific islander. ^a^ Persistent poverty was defined as when 20% or more of the population has lived below the poverty level for a period spanning about 30 years. Rurality was defined by using the U.S. Department of Agriculture’s (USDA) Rural Urban Commuting Area (RUCA) codes. RUCA codes 1.0, 1.1, 2.0, 2.1, 3.0, 4.1, 5.1, 7.1, 8.1, and 10.1 were considered as urban areas (termed as no) and all other codes were classified as rural areas (termed as yes). ^b^ Other includes divorced, separated, and widow. ^c^ Grade 1: well-differentiated; Grade 2: moderately differentiated; Grade 3: poorly differentiated; Grade 4: undifferentiated. ^d^ Right: cecum to transverse; left: splenic flexure to rectum.

**Table 2:** Results from proportional hazards models. Association between race/ethnicity, persistent poverty, and rurality on the risk of CRC death.

	Crude Model ^a^		Model 1 ^a^		Model 2 ^a^		Model 3 ^a^	
	HR (95% CI)	*p*-Value	HR (95% CI)	*p*-Value	HR (95% CI)	*p*-Value	HR (95% CI)	*p*-Value
**Race/ethnicity**		<0.001		<0.001		<0.001		<0.001
NHW	Reference		Reference		Reference		Reference	
NHB	1.13 (1.11, 1.15)		1.20 (1.18, 1.22)		1.16 (1.14, 1.18)		1.12 (1.10, 1.13)	
AI/AN	1.02 (0.95, 1.09)		1.11 (1.03, 1.19)		1.09 (1.02, 1.17)		1.05 (0.97, 1.12)	
Asian/PI	0.84 (0.82, 0.86)		0.92 (0.90, 0.94)		0.92 (0.90, 0.94)		0.91 (0.89, 0.93)	
Hispanic	0.95 (0.93, 0.96)		1.04 (1.02, 1.05)		1.00 (0.98, 1.01)		0.97 (0.96, 0.99)	
**Persistent poverty** ^b^		<0.001		<0.001		<0.001		<0.001
No	Reference		Reference		Reference		Reference	
Yes	1.15 (1.13, 1.16)		1.12 (1.10, 1.14)		1.11 (1.09, 1.13)		1.10 (1.08, 1.12)	
**Rurality** ^b^		<0.001		<0.001		<0.001		<0.001
No	Reference		Reference		Reference		Reference	
Yes	1.07 (1.05, 1.08)		1.09 (1.07, 1.10)		1.10 (1.09, 1.12)		1.11 (1.10, 1.13)	

Abbreviations: NA, non-applicable; CRC, colorectal cancer; HR, hazard ratio; NHW, non-Hispanic White; NHB, non-Hispanic Black; AI/AN, American Indian/Alaska Native; PI, Pacific Islander. Italicized text indicates statistically significant result. ^a^ Crude models included race/ethnicity, persistent poverty, and rurality; model 1 was further adjusted for demographic characteristics (age, gender, marital status) and year of diagnosis; model 2 was further adjusted for tumor characteristics (grade, SEER summary stage, primary site); model 3 was further adjusted for treatment modality (surgery, radiation, chemotherapy). ^b^ Persistent poverty was defined as when 20% or more of the population has lived below the poverty level for a period spanning about 30 years. Rurality was defined by using the U.S. Department of Agriculture’s (USDA) Rural Urban Commuting Area (RUCA) codes. RUCA codes 1.0, 1.1, 2.0, 2.1, 3.0, 4.1, 5.1, 7.1, 8.1, and 10.1 were considered as urban areas (termed as no) and all other codes were classified as rural areas (termed as yes).

**Table 3:** Predictors of CRC death. Intersectional relationship of persistent poverty and rurality on the risk of CRC death.

	Rurality ^b^ No		Rurality ^b^ Yes		
	5-Year CRC Survival Status^c^ No/Yes n (%)	HR (95% CI) ^a^	5-Year CRC Survival Status ^c^ No/Yes n (%)	HR (95% CI) ^a^	HR (95% CI) for Rurality Within Strata of Poverty
**Persistent poverty ^b^**					
**Non-Hispanic White**					
No (n = 343,676)	90,702 (31.2%)/199,828 (66.8%)	Reference	17,491 (32.9%)/35,655 (67.1%)	1.10 (1.08,1.12)	1.10 (1.08, 1.12)
Yes (n = 19,829)	4160 (35.4%)/7589 (64.6%)	1.13 (1.10, 1.17)	2819 (34.9%)/5261 (65.1%)	1.26 (1.21,1.31)	1.12 (1.06, 1.17)
HR (95% CI) for poverty within strata of rurality	1.13 (1.10, 1.17)		1.15 (1.10, 1.19)		
**Non-Hispanic Black**					
No (n = 45,843)	14,859 (34.7%)/27,936 (65.3%)	Reference	1145 (37.6%)/1903 (62.4%)	1.11 (1.05, 1.18)	1.11 (1.05, 1.18)
Yes (n = 17,694)	5859 (38.1%)/9517 (61.9%)	1.10 (1.07, 1.14)	901 (38.9%)/1417 (61.1%)	1.20 (1.12, 1.28)	1.09 (1.01, 1.16)
HR (95% CI) for poverty within strata of rurality	1.10 (1.07, 1.14)		1.07 (0.98, 1.17)		
**AI/AN**					
No (n = 1782)	425 (31.6%)/922 (68.5%)	Reference	157 (36.1%)/278 (63.9%)	1.19 (0.98, 1.43)	1.19 (0.98, 1.43)
Yes (n = 454)	75 (31.5%)/163 (68.5%)	0.99 (0.77, 1.27)	93 (43.1%)/123 (56.9%)	1.66 (1.32, 2.08)	1.67 (1.23, 2.28)
HR (95% CI) for poverty within strata of rurality	0.99 (0.77, 1.27)		1.40 (1.08, 1.82)		
**Asian/PI**					
No (n = 33,113)	8787 (27.4%)/23,264 (72.6%)	Reference	338 (31.8%)/724 (68.2%)	1.17 (1.05, 1.31)	1.17 (1.05, 1.31)
Yes (n = 2340)	721 (32.5%)/1501 (67.6%)	1.11 (1.03, 1.19)	38 (32.2%)/80 (67.8%)	1.19 (0.87, 1.64)	1.08 (0.78, 1.50)
HR (95% CI) for poverty within strata of rurality	1.11 (1.03, 1.19)		1.02 (0.73, 1.42)		
**Hispanic**					
No (n = 52,460)	14,959 (30.6%)/33,887 (69.4%)	Reference	1219 (33.7%)/2395 (66.3%)	1.15 (1.09, 1.22)	1.15 (1.09, 1.22)
Yes (n = 15,677)	4546 (32.6%)/9419 (67.5%)	1.05 (1.01, 1.09)	586 (34.2%)/1126 (65.8%)	1.18 (1.09, 1.29)	1.12 (1.03, 1.23)
HR (95% CI) for poverty within strata of rurality	1.05 (1.02, 1.09)		1.03 (0.93, 1.14)		

Abbreviations: CRC, colorectal cancer; HR, hazard ratio; NHW, non-Hispanic White; NHB, non-Hispanic Black; AI/AN, American Indian/Alaska Native; PI, Pacific Islander. Italicized text indicates statistically significant result. ^a^ All models were adjusted for demographic characteristics (age, gender, marital status), year of diagnosis, tumor characteristics (grade, SEER summary stage, primary site), and treatment modality (surgery, radiation, chemotherapy), including NHW, NHB, AI/AN, Asian/PI, and Hispanic patients. *p*-values for multiplicative interactions are <0.05 for NHW (<0.001), NHB (<0.001), Hispanic (<0.001), Asian/PI (0.002), and AI/AN (<0.001). ^b^ Persistent poverty was defined as when 20% or more of the population has lived below the poverty level for a period spanning about 30 years. Rurality was defined by using the U.S. Department of Agriculture’s (USDA) Rural Urban Commuting Area (RUCA) codes. RUCA codes 1.0, 1.1, 2.0, 2.1, 3.0, 4.1, 5.1, 7.1, 8.1, and 10.1 were considered as urban areas (termed as no) and all other codes were classified as rural areas (termed as yes). ^c^ Patients’ 5-year CRC survival status (no or yes) were provided using row percentage.

## Data Availability

The data used for this study are available from the U.S. National Cancer Institute website.

## Supplementary Materials

The following supporting information can be downloaded at: https://www.mdpi.com/article/10.3390/curroncol32050248/s1. Supplementary Table S1. Results from proportional hazards models. Association between race/ethnicity, persistent poverty, and rurality on the risk of CRC death (unknown tumor grade and SEER summary stage were removed).

## Appendix A. STROBE Statement—Checklist of Items That Should Be Included in Reports of *Cohort Studies*

	**Item No**	**Recommendation**	**Page No**
**Title and abstract**	1	(*a*) Indicate the study’s design with a commonly used term in the title or the abstract	
		(*b*) Provide in the abstract an informative and balanced summary of what was done and what was found	1
**Introduction**			
Background/rationale	2	Explain the scientific background and rationale for the investigation being reported	1, 2
Objectives	3	State specific objectives, including any prespecified hypotheses	2
**Methods**			
Study design	4	Present key elements of study design early in the paper	1, 2
Setting	5	Describe the setting, locations, and relevant dates, including periods of recruitment, exposure, follow-up, and data collection	2
Participants	6	(*a*) Give the eligibility criteria, and the sources and methods of selection of participants. Describe methods of follow-up	
		(*b*) For matched studies, give matching criteria and number of exposed and unexposed	2, 3
Variables	7	Clearly define all outcomes, exposures, predictors, potential confounders, and effect modifiers. Give diagnostic criteria, if applicable	3
Data sources/measurement	8 *	For each variable of interest, give sources of data and details of methods of assessment (measurement). Describe comparability of assessment methods if there is more than one group	2, 3
Bias	9	Describe any efforts to address potential sources of bias	3, 4
Study size	10	Explain how the study size was arrived at	2, 3
Quantitative variables	11	Explain how quantitative variables were handled in the analyses. If applicable, describe which groupings were chosen and why	3
Statistical methods	12	(*a*) Describe all statistical methods, including those used to control for confounding	
		(*b*) Describe any methods used to examine subgroups and interactions	
		(*c*) Explain how missing data were addressed	3, 4
		(*d*) If applicable, explain how loss to follow-up was addressed	
		(*e*) Describe any sensitivity analyses	
**Results**			
Participants	13 *	(*a*) Report numbers of individuals at each stage of study—eg numbers potentially eligible, examined for eligibility, confirmed eligible, included in the study, completing follow-up, and analysed	2, 3
		(*b*) Give reasons for non-participation at each stage	
		(*c*) Consider use of a flow diagram	
Descriptive data	14 *	(*a*) Give characteristics of study participants (eg demographic, clinical, social) and information on exposures and potential confounders	
		(*b*) Indicate number of participants with missing data for each variable of interest	4, 5
		(*c*) Summarise follow-up time (eg, average and total amount)	
Outcome data	15 *	Report numbers of outcome events or summary measures over time	5, 6
Main results	16	(*a*) Give unadjusted estimates and, if applicable, confounder-adjusted estimates and their precision (eg, 95% confidence interval). Make clear which confounders were adjusted for and why they were included	
		(*b*) Report category boundaries when continuous variables were categorized	4, 9
		(*c*) If relevant, consider translating estimates of relative risk into absolute risk for a meaningful time period	
Other analyses	17	Report other analyses done—eg analyses of subgroups and interactions, and sensitivity analyses	9
**Discussion**			
Key results	18	Summarise key results with reference to study objectives	9
Limitations	19	Discuss limitations of the study, taking into account sources of potential bias or imprecision. Discuss both direction and magnitude of any potential bias	10,11
Interpretation	20	Give a cautious overall interpretation of results considering objectives, limitations, multiplicity of analyses, results from similar studies, and other relevant evidence	10
Generalisability	21	Discuss the generalisability (external validity) of the study results	10
**Other information**			
Funding	22	Give the source of funding and the role of the funders for the present study and, if applicable, for the original study on which the present article is based	11
* Give information separately for exposed and unexposed groups. **Note:** An Explanation and Elaboration article discusses each checklist item and gives methodological background and published examples of transparent reporting. The STROBE checklist is best used in conjunction with this article (freely available on the Web sites of PLoS Medicine at http://www.plosmedicine.org/, Annals of Internal Medicine at http://www.annals.org/, and Epidemiology at http://www.epidem.com/). Information on the STROBE Initiative is available at http://www.strobe-statement.org (accessed on 20 April 2025).		
