# Supplementary material for: The Influence of Poverty and Rurality on Colorectal Cancer Survival by Race/Ethnicity: An Analysis of SEER Data with a Census Tract-Level Measure of Persistent Poverty
Source: Curr Oncol. 2025 Apr 23;32(5):248. doi: 10.3390/curroncol32050248 (PMC12109587; doi:10.3390/curroncol32050248)
Supplement: Supplementary file 1 [file curroncol-32-00248-s001.zip › curroncol-3560916-supplementary.pdf]

Supplementary Table S1. Results from proportional hazards models. Association between race/ethnicity, persistent poverty, and rurality on the risk of CRC death (unknown tumor grade and SEER summary stage were removed)

	**Full Model [a]**		**Reduced model [a]**	
	**HR (95%CI)**	**P-value**	**HR (95%CI)**	**P-value**
**Race/ethnicity**		<0.001		<0.001
NHW	Reference		Reference	
NHB	*1.12 (1.10,1.13)*		*1.18(1.16,1.20)*	
AI/AN	1.05 (0.97,1.12)		1.05(0.97,1.14)	
Asian/PI	*0.91(0.89,0.93)*		*0.93(0.91,0.95)*	
Hispanic	0.97 (0.96,0.99)		0.99(0.98,1.01)	
**Persistent poverty [b]**		<0.001		<0.001
No	Reference		Reference	
Yes	*1.10(1.08,1.12)*		1.11(1.09,1.13)	
**Rurality [b]**		<0.001		<0.001
No	Reference		Reference	
Yes	*1.11 (1.10,1.13)*		*1.11(1.09,1.13)*	

Abbreviations: NA, non-applicable; CRC, colorectal cancer; HR, hazard ratio; NHW, non-Hispanic White; NHB, non-Hispanic Black; AI/AN, American Indian/Alaska Native; PI, Pacific Islander.

Italicized text indicates statistically significant result.

[a] Full model included unknown tumor grade and SEER summary stage; reduced model did not include unknown tumor grade and SEER summary stage. Both models were adjusted for all covariates (demographic characteristics, year of diagnosis, tumor characteristics, and treatment modality.

[b] Persistent poverty was defined as when 20% or more of the population has lived below the poverty level for a period spanning about 30 years. Rurality was defined by using the U.S. Department of Agriculture's (USDA) Rural Urban Commuting Area (RUCA) codes. RUCA codes 1.0, 1.1, 2.0, 2.1, 3.0, 4.1, 5.1, 7.1, 8.1, and 10.1 were considered as urban areas (termed as no) and all other codes were classified as rural areas (termed as yes).
